# 4-Pyridone-3-carboxamide-1-β-D-ribonucleoside (4PYR)—A Novel Oncometabolite Modulating Cancer-Endothelial Interactions in Breast Cancer Metastasis

**DOI:** 10.3390/ijms23105774

**Published:** 2022-05-21

**Authors:** Patrycja Koszalka, Barbara Kutryb-Zajac, Paulina Mierzejewska, Marta Tomczyk, Joanna Wietrzyk, Pawel K. Serafin, Ryszard T. Smolenski, Ewa M. Slominska

**Affiliations:** 1 Institute of Medical Biotechnology and Experimental Oncology, Intercollegiate Faculty of Biotechnology UG-MUG, Medical University of Gdansk, 80-210 Gdansk, Poland; pserafin@gumed.edu.pl; 2 Department of Biochemistry, Medical University of Gdansk, 80-210 Gdansk, Poland; b.kutryb-zajac@gumed.edu.pl (B.K.-Z.); paulina.zukowska@gumed.edu.pl (P.M.); marta.tomczyk@gumed.edu.pl (M.T.); rt.smolenski@gumed.edu.pl (R.T.S.); 3 Hirszfeld Institute of Immunology and Experimental Therapy, Polish Academy of Sciences, 53-114 Wroclaw, Poland; wietrzyk@iitd.pan.wroc.pl

**Keywords:** 4-pyridone-3-carboxamide-1-β-D-ribonucleoside, breast cancer, adenine nucleotides, endothelial cells, lung metastasis

## Abstract

The accumulation of specific metabolic intermediates is known to promote cancer progression. We analyzed the role of 4-pyridone-3-carboxamide-1-β-D-ribonucleoside (4PYR), a nucleotide metabolite that accumulates in the blood of cancer patients, using the 4T1 murine in vivo breast cancer model, and cultured cancer (4T1) and endothelial cells (ECs) for in vitro studies. In vivo studies demonstrated that 4PYR facilitated lung metastasis without affecting primary tumor growth. In vitro studies demonstrated that 4PYR affected extracellular adenine nucleotide metabolism and the intracellular energy status in ECs, shifting catabolite patterns toward the accumulation of extracellular inosine, and leading to the increased permeability of lung ECs. These changes prevailed over the direct effect of 4PYR on 4T1 cells that reduced their invasive potential through 4PYR-induced modulation of the CD73-adenosine axis. We conclude that 4PYR is an oncometabolite that affects later stages of the metastatic cascade by acting specifically through the regulation of EC permeability and metabolic controls of inflammation.

## 1. Introduction

4PYR (4-pyridone-3-carboxamide-1-β-D-ribonucleoside, PCNR, ribosylpyridin-4-one-3-carboxamide), a naturally occurring pyridine nucleoside, can be detected in healthy individuals in both plasma and urine within a nanomolar range [1]. However, for many pathological states, including chronic myelogenous leukemia, and lung and breast cancer, a significant increase in its concentration has been demonstrated [2,3,4] in correlation with disease progression [5].

4PYR is functionally and metabolically related to nicotinamide (NA, niacinamide), a precursor or constituent of many compounds, including nicotinamide adenine dinucleotide (NAD^+^). 4PYR is primarily produced in the liver by the cytosolic aldehyde oxidase (AO)-regulated oxidation of nicotinamide riboside (NR) [6,7]. AO is expressed to a relatively low extent in other organs, but its expression and activity are prone to upregulation [8,9]. Furthermore, NR can also be generated by CD73 (ecto-5′-nucleotidase, eNT), a key regulator in cancer metastasis [10] that is overexpressed in many types of murine and human cancer cells [11], including breast cancer (BC) [12]. CD73 is a crucial enzyme in the extracellular adenosine (eAdo)-generating cascade, mainly catalyzing 5′-AMP conversion to adenosine [13], and thus regulating eAdo signaling through four G-protein-coupled adenosine receptors (A_1_, A_2A_, A_2B_, and A_3_) [14]. However, CD73 can also process NAD^+^ into adenosine and NR [15,16]. Adenosine kinase phosphorylates 4PYR to its metabolic by-products, mono-, di-, and triphosphates (4PYMP, 4PYDP, and 4PYTP), or its NAD^+^ analog (4PYR adenylate diphosphate: 4PYRAD). The rapid metabolization of 4PYR can be cytotoxic for many tumor and endothelial cells, via a decrease in ATP and NAD^+^ levels [17,18,19].

Recently, the abnormal accumulation of metabolic intermediates emerged as a potential new driving force in metastasis. This multi-step process is an ongoing challenge in the treatment of human malignancy, and is the leading cause of cancer-related death, especially in breast cancer (BC) [20]. Oncometabolites can modulate the invasive potential of tumor cells, their survival during dissemination, and their adaptation during the colonization stage. Their connection with the inflammatory state of the tumor microenvironment was also pointed out [21,22].

In this study, our goal was to define the role of 4PYR in the metastatic process, using a model of a highly invasive murine breast cancer cell line, 4T1, with a triple-negative molecular profile that is syngeneic to the BALB/c mouse strain, and with a similarly aggressive phenotype to the human disease [23]. We demonstrated that 4PYR could enhance metastasis formation through the increased permeabilization of the lung endothelium and an accumulation of anti-inflammatory inosine. We have also demonstrated a correlation between the 4PYR and CD73-adenosine axis, which was recently indicated as being one of the crucial regulators for cancer invasive potential [24]. Our results support the premise of 4PYR as being a novel oncometabolite at the later stages of the metastatic process.

## 2. Results

### 2.1. 4PYR Enhances Lung Metastasis Formation but Does Not Affect Primary Tumor Growth

**Figure 1:**
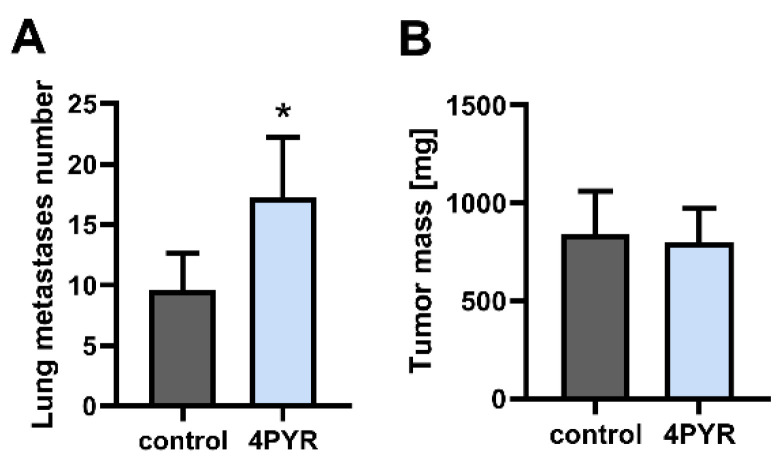
4PYR increases lung metastasis formation but does not affect tumor growth in the 4T1 breast cancer model. The comparison between 4PYR-treated mice and saline-treated control in the number of lung metastases (**A**) and tumor mass (**B**). Lung metastases were analyzed in the 4T1 experimental metastasis model (1.5 × 10^4^ of 4T1 cells injected intravenously), and tumor mass, in the 4T1 orthotopic model (3 × 10^5^ of 4T1 cells injected into a mammary fad pad). Mice were euthanized 28 days after the injection of 4T1 cells. The number of surface metastatic loci was counted with a stereomicroscope, and confirmed using histological analysis. The tumor was weighted. In both models, 4PYR or the saline (control) was administered subcutaneously every 12 h (100 mg/kg/24 h) from the moment of the injection of the 4T1 cells. The graphs represent the mean ± SEM from two independent experiments, *n* = 10 per group. * *p* < 0.05.

In the experimental metastasis model that was used to evaluate the extravasation and organ colonization stage of the metastatic cascade [25], we simulated 4PYR accumulation in the blood plasma observed during cancer progression [5]. BALB/c mice that were injected intravenously with 4T1 cells were treated with 4PYR at a concentration that was needed to obtain comparable levels of its triphosphate derivatives, and 4PYRAD to these in oncological patients [1]. 4PYR induced a significant increase in the number of lung metastases, a 79% increase compared to the control (treated with 0.9% NaCl) (*p* < 0.05) at the experimental endpoint (the 28th day from the injection of the 4T1 cells) (Figure 1A).

As a next step, we analyzed whether 4PYR can affect the growth of a primary tumor in an orthotopic breast cancer model. BALB/c mice were injected into the mammary fat pad with 4T1 cells and treated with 4PYR. At the experimental endpoint (the 28th day from the injection with 4T1 cells), 4PYR had no significant effect on the tumor mass (Figure 1B).

Therefore, 4PYR accumulation during breast cancer progression in vivo seems to have a pro-metastatic effect in the later stage of metastatic progression during lung metastases development, in agreement with its significant accumulation in patients with metastatic BC [4].

### 2.2. 4PYR Inhibits the Invasive Potential of 4T1 Breast Cancer Cells In Vitro

**Figure 2:**
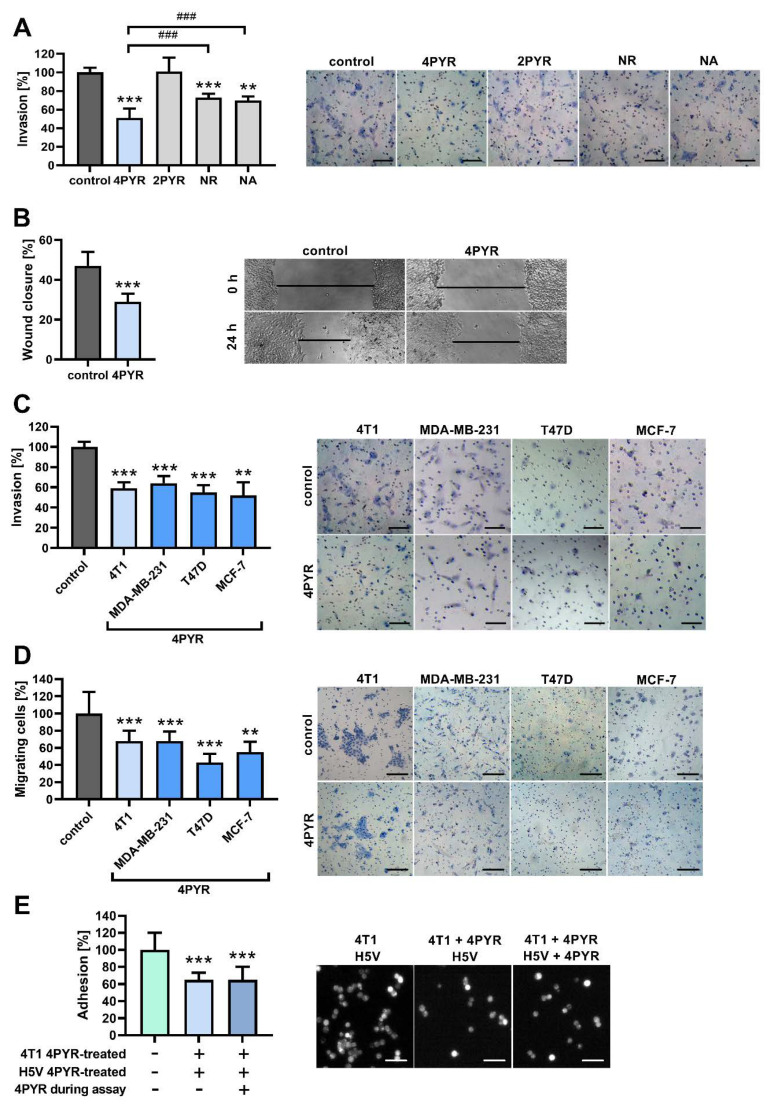
4PYR decreases the invasion and migration of human and murine breast cancer cell lines in vitro. (**A**) Transwell invasion assay of 4T1 cells treated with 4PYR, 2PYR, NR, or NA (100 μM, each), or vehicle (control). Cells that invaded through Matrigel in the Transwell invasion chamber after 24 h were stained with Mayer’s Hematoxylin and counted under the microscope. Representative images are shown. Bars, 100 me. (**B**) Wound healing assay of 4T1 cells treated with 4PYR (100 μM) compared to vehicle-treated control. The wound closure was calculated by subtracting the width of the wound 24 h after its initial width at time 0 (demonstrated as bars on representative images). (**C**,**D**) Transwell invasion assay (**C**) or Transwell migration assay (**D**) of murine 4T1 cells and human BC cell lines (MDA-MB-231, T47D, and MCF-7) treated with 4PYR (100 μM) compared to vehicle-treated control. Cells that invaded Matrigel/migrated through the filter after 24 h were stained with Mayer’s Hematoxylin and counted under the microscope. Representative images are shown. Bars, 100 μm. (**E**) Static phase adhesion assay analyzing 4T1 cell adherence to a monolayer of H5V endothelial cells. Where indicated on a graph (+4PYR), cells were treated with 100 μM 4PYR overnight before the assay or during the assay. Adherent 4T1 cells labeled with Calcein AM were counted after 15 min under an epifluorescence microscope. Representative images are shown. Bars, 50 μm. Graphs represent the mean ± SEM from four independent experiments. ** *p* < 0.01, *** *p* < 0.001 vs. control, ^###^ *p* < 0.001 when comparison was performed between two other groups.

As a next step, we tested whether 4PYR can impact the invasive potential of tumor cells in vitro. In the Transwell invasion assay, 4PYR decreased the invasion of 4T1 cells through Matrigel (ECM from EHS sarcoma) down to 59% of the control (*p* < 0.001) (Figure 2A). In the wound healing assay designed to analyze the collective migration of cells, 4PYR decreased wound closure down to 62% of the control (*p* < 0.001) (Figure 2B). These effects were dose-dependent (Figure S1A,B). The viability of the 4T1 cells was not affected by up to 400 μM of 4PYR (Figure S2A).

4PYR has two minor structural isomers, with 2PYR (2-pyridone-5-carboxamide-1-D-ribonucleoside) being the more prominent isomer [26]. 2PYR did not affect the invasion of 4T1 cells through Matrigel (Figure 2A), indicating 4PYR as being an active derivative of NR. However, the supplementation of NA, a precursor of NR, was shown to decrease tumor growth and the number of cancer nodules in the liver in rat hepatocellular carcinoma [27]. We observed that while both NR and NA had some inhibitory effect on the invasive potential of 4T1 cells in vitro, this effect was significantly weaker than an effect that was induced by 4PYR (by 22% and 19%, respectively, *p* < 0.001) (Figure 2A). Thus, the 4T1 murine cell line invasive potential is inhibited mainly by 4PYR, and not by its precursors.

Next, we analyzed whether the effect of 4PYR on the BC cells in vitro can be translated into the human model, as some metabolic effects induced by 4PYR were cell-specific and possibly organ- or species-specific [17,19]. The Transwell invasion assay (Figure 2C) and wound healing assay (Figure 2D) gave comparable results between 4T1 and a variety of human BC cell lines according to their histological and molecular subtypes, as well as their invasive potential: MDA-MB-231 (BasalB, triple-negative adenocarcinoma without amplified EGFR, highly invasive), T47D (Luminal, ER- and PR-positive invasive ductal carcinoma without HER2 amplification, with middle–low invasive potential) and MCF-7 (Luminal, ER- and PR-positive invasive ductal carcinoma without HER2 amplification, with low invasive potential). This indicates a ubiquitous effect of 4PYR on the tumor cells’ invasive phenotype.

Tumor cell–endothelium interactions are a crucial factor in cancer metastasis [28]. In the static adhesion assay [29], 4PYR decreased, down to 65% of the control (*p* < 0.001) (Figure 2E), an attachment of 4T1 breast cancer cells to a monolayer formed by the H5V cell line (murine immortalized endothelial cells (ECs) from the heart microvessels of C57BL/6 mice). The effect was similar when only 4T1 cells were pretreated with 4PYR, and when both tumor and EC cells were treated with 4PYR, indicating that 4PYR affects mainly tumor cells within this process. The viability of the H5V cells was not affected by up to 200 μM of 4PYR (Figure S2C).

### 2.3. 4PYR-Induced Modulation of the Invasive Potential of 4T1 Cells Is Dependent on CD73 Activity and Mediated through Adenosine Receptors

**Figure 3:**
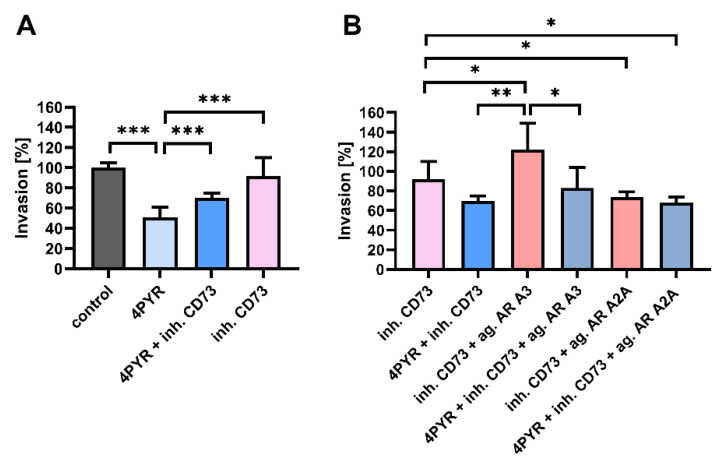
The effect of 4PYR on the invasive potential of 4T1 cells is blocked by CD73 inhibition and mediated through adenosine receptors (AR). (**A**) Transwell invasion assay of 4T1 cells treated with 4PYR (100 μM), CD73 inhibitor, AOPCP (200 μM; inh. CD73), or their combination, relative to vehicle-treated control. (**B**) Transwell invasion assay of 4T1 cells treated with 4PYR (100 μM), specific AR agonists (ag. AR A3, IB-MECA for A_3_AR; ag. AR A2A, CGS-21680 for A_2A_AR; 10 μM of each agonist), or their combination in the presence of CD73 inhibitor (to inhibit endogenous eAdo generation), relative to vehicle-treated control (100%; the bar is not shown). Cells that invaded through Matrigel in the Transwell invasion chamber after 24 h were stained with Mayer’s Hematoxylin and counted under the microscope. The graph represents the mean ± SEM from three independent experiments. * *p* < 0.05, ** *p* < 0.01, *** *p* < 0.001.

Some changes in the invasive potential of tumor cells induced by 4PYR were similar to the changes induced via the inhibition of CD73, e.g., their decreased rate of migration [30] and an attachment to the EC in vitro [31]. We have, therefore, analyzed the connection between CD73 activity and the 4PYR effect on the invasive potential of the 4T1 cells. A CD73 competitive inhibitor, AOPCP (adenosine 5′-(α,β-methylene)diphosphate), effectively reversed the 4PYR-induced inhibition of ECM invasion by 4T1 cells (Figure 3A). By itself, AOPCP did not affect this process.

Next, we analyzed whether these changes can be mediated through adenosine receptors (AR), as NAD^+^ was shown to act as an A_2A_AR-selective agonist [32]. We have also demonstrated that the activation of A_2A_AR and A_3_AR can modulate the invasive potential of the 4T1 cells [33]. To stimulate single adenosine receptors, we used selective AR agonists (CGS-21680 for the A_2A_ adenosine receptor, IB-MECA for the A_3_ adenosine receptor) in the presence of AOPCP to inhibit endogenous eAdo production. The stimulation of A_3_AR increased ECM invasion (by 30%, *p* < 0.05), while the stimulation of A_2A_AR decreased it (a decrease of 18%, *p* < 0.05) (Figure 3B). 4PYR was able to reverse the effect of A_3_AR activation (*p* < 0.01) without affecting the outcome of A_2A_AR activation. Thus, 4PYR can regulate the invasive potential of tumor cells through adenosine receptors, mainly A_3_AR.

### 2.4. 4PYR Up-Regulates AMP Hydrolysis and Adenosine Deamination during Paracrine Interactions between Cancer and Endothelial Cells, Leading to a Significant Accumulation of Extracellular Inosine

4PYR can also modulate the activity of extracellular enzymes involved in adenosine generation, including an increase in CD73 activity on endothelial cells [18]. Pro-inflammatory extracellular ATP (eATP) is one of the crucial regulators of the tumor-induced activation of EC [34], as cancer cells can increase its basal release using EC via cytokines, such as TNF-α (tumor necrosis factor-alpha) [35,36]. However, eATP is rapidly hydrolyzed to eAdo, whose significant accumulation triggers its irreversible deamination to anti-inflammatory extracellular inosine (eIno) [37].

**Figure 4:**
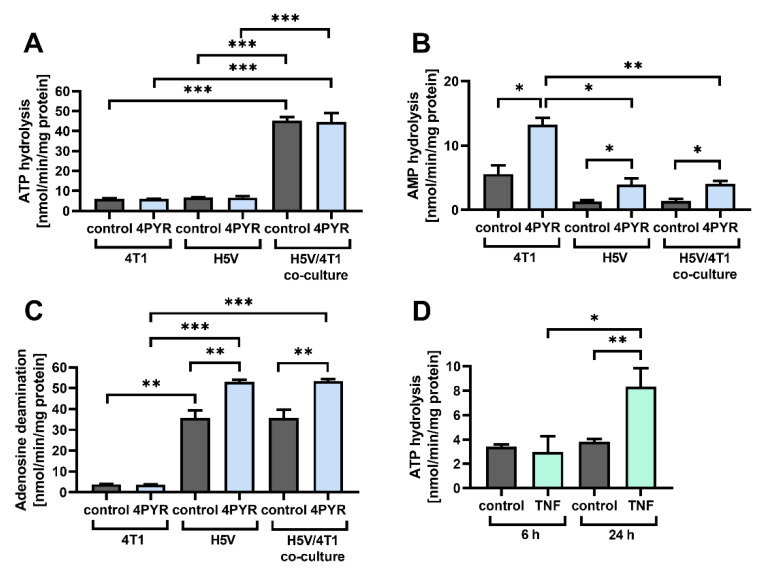
4PYR regulates eAdo generation and deamination during paracrine interactions between 4T1 and H5V endothelial cells. (**A**–**C**) Effect of 4PYR (100 μM) on the rate of extracellular ATP hydrolysis (**A**), AMP hydrolysis (**B**), and adenosine deamination (**C**) in the co-culture of H5V and 4T1 cells, compared to single-cell type cultures. Vehicle-treated cells were used as a control. Cells were separated using a filter (pore size of 1 µm) in a Transwell chamber. 4PYR was added to the upper chamber containing 4T1 cells. Enzymatic activity was measured via reversed-phase HPLC after 72 h of culture. (**D**) Effect of TNF (10 ng/mL) on the rate of eATP hydrolysis by H5V cells. Enzymatic activity was measured on 96-well plates via reversed-phase HPLC after 6 or 24 h of culture. Vehicle-treated cells were used as a control. Graphs represent the mean ± SEM from four independent experiments. * *p* < 0.05, ** *p* < 0.01, *** *p* < 0.001.

Therefore, we analyzed whether 4PYR can modulate extracellular adenine nucleotide metabolism during paracrine interactions between 4T1 tumor cells and H5V endothelial cells. A co-culture incubation time of 72 h in the Transwell chamber induced a significant increase in ATP hydrolysis (approximately 7×, *p* < 0.001), a change that was not modulated by 4PYR (Figure 4A). However, 4PYR had a significant effect on AMP hydrolysis to adenosine, upregulating it in H5V/4T1 co-culture as well in 4T1 and H5V single-cell type cultures (*p* < 0.05) (Figure 4B). It also significantly upregulated adenosine deamination to inosine in both the H5V/4T1 co-culture and the H5V cell culture (*p* < 0.01), without affecting the low adenosine deaminase activity in the 4T1 cell culture (Figure 4C).

TNF-α stimulation of H5V cells induced a similar increase in the rate of ATP hydrolysis (Figure 4D) as their co-culture with 4T1 cells. This indicates a higher degree of pro-inflammatory ATP release through paracrine loops between the tumor and the EC cells. The resulting eAdo accumulation can be enhanced by 4PYR, but a 4PYR-induced increase in eAdo deamination rate might change the balance in favor of inosine-mediated changes.

### 2.5. 4PYR Can Induce Contradictory Alterations in the Barrier Function of Endothelial Cells in Correlation with Differences in Their Extracellular Adenine Nucleotide Metabolism, Energy Status, and Oxidoreductive Potential

The structural integrity of an endothelial barrier is a crucial factor in cancer cell extravasation [38]. eAdo is one of its main regulators, promoting the resealing of the endothelial junctions through adenosine receptor-modulated changes in cAMP levels [39]. 4PYR can upregulate both eAdo generation and deamination, as well as modulating AR signaling. Thus, we have analyzed whether it can affect the permeability of an endothelial layer in vitro. However, endothelial cells from different vascular beds demonstrate high levels of heterogeneity during homeostasis and inflammation [40]. Therefore, we have compared the differences in the extracellular activities of enzymes that are involved in adenine nucleotide catabolism between H5V cells and lung endothelial cells (LECs) isolated from C57BL/6 mice.

**Figure 5:**
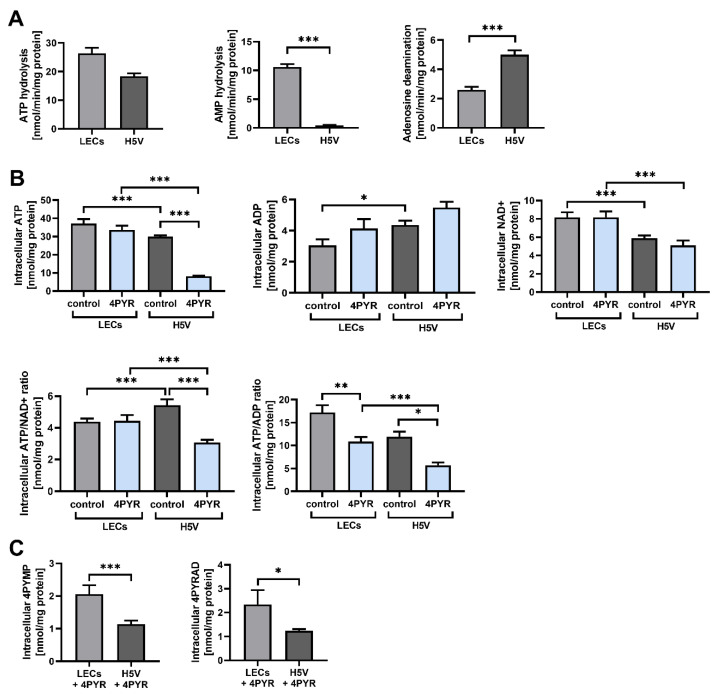
Heterogeneity in the energy status, oxidoreductive potential, and 4PYR degradation ratio between LECs and H5V cells. (**A**) Comparison between the rate of eATP hydrolysis, eAMP hydrolysis, and eAdo deamination in LECs and H5V cell cultures. Enzymatic activities were measured via reversed-phase HPLC after 24 h of culture. Graphs represent the mean ± SEM from six independent experiments. *** *p* < 0.001. (**B**,**C**) Effect of 4PYR (100 μM) on the intracellular concentration of nucleotides and their catabolites (**B**), and 4PYR metabolites (**C**) in LECs and H5V cells, relative to vehicle-treated control. Cell extracts were analyzed via reversed-phase HPLC after 72 h of cell culture. Protein concentration was measured with the Bradford method in protein precipitates. Graphs represent normalized averages ± SEM from six independent experiments. * *p* < 0.05, ** *p* < 0.01, *** *p* < 0.001.

The H5V cells and LECs showed no significant differences in their rates of ATP hydrolysis, but LECs had a higher rate of AMP hydrolysis compared to eAdo (a 96% increase, *p* < 0.001), and a decreased rate of eAdo deamination compared to inosine (48%, *p* < 0.001) (Figure 5A). This indicates a higher rate of eAdo accumulation in LECs, promoting tight junction formation. Such a highly restrictive barrier for the paracellular route of extravasation is characteristic of lung endothelium [41].

However, tumor cells adopt a strategy to induce the foci of vascular hyperpermeability in lung ECs [42], a process that is measured as an albumin flux and that is highly dependent on the EC energy status [43]. Compared to the H5V cell line, LECs had a significantly higher cell energy status, with a distinctly elevated intracellular ATP (iATP) reserve (an increase of 40%, *p* < 0.001) and a comparably decreased intracellular ADP (iADP) concentration (an increase of 42%, *p* < 0.05) (Figure 5B). Treatment with 4PYR for 72 h had induced no significant changes in the iATP pool in LECs, while it strongly depleted smaller iATP reserves in H5V cells (an increase of 73% compared to the control, *p* < 0.001). 4PYR induced no significant changes in the iADP level. LECs also had higher intracellular NAD^+^ (iNAD^+^) reserves (an increase of 66%, *p* < 0.001) compared to H5V cells, indicating a higher oxidoreductive potential. However, 4PYR did not affect the iNAD^+^ pool.

A higher iATP pool in LECs correlated with a higher accumulation of 4PYR metabolic by-products, 4PYMP and 4PYRAD, compared to H5V cells (an increase of 45%, *p* < 0.05, and 47%, *p* < 0.001, respectively) (Figure 5C). This suggests a faster rate of 4PYR metabolization by LECs without any significant deterioration of their energy status, unlike H5V cells, which demonstrated a tremendous loss in iATP level.

**Figure 6:**
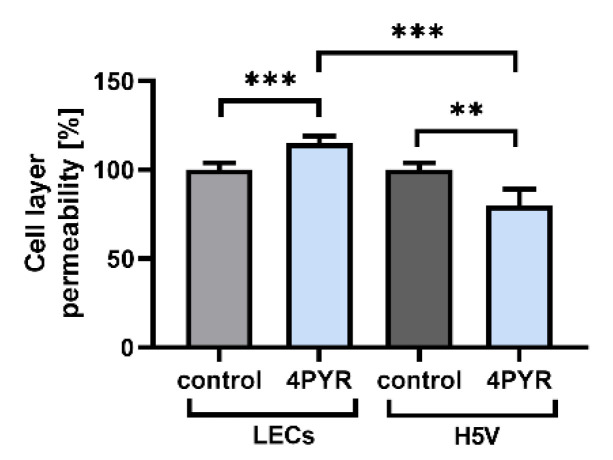
Divergent regulation of endothelial permeability by 4PYR. Effect of 4PYR on the permeability of LECs or H5V monolayers. Permeability was measured in a Transwell chamber (1 µm pore size) as a flux of BSA-bound Evans blue dye 22 h after the addition of 100 μM of 4PYR to the upper chamber containing ECs. Monolayer integrity was assessed using staining with Mayer’s Hematoxylin. Any inserts with cell detachment were discarded from the data set. The graph represents the mean ± SEM from four independent experiments. ** *p* < 0.01, *** *p* < 0.001 vs. control (non-treated cells).

Next, we evaluated whether 4PYR can induce divergent effects on the endothelial layer permeability of LECs and H5V monolayers, by analyzing the flux of BSA-bound Evans blue dye (approximately 67.5 kDa) in the Transwell chamber [42,44]. After 22 h of incubation, 4PYR decreased the permeability of the H5V cell line monolayer by 20% (*p* < 0.01), but increased the permeability of the LECs monolayer by 15% (*p* < 0.001) as compared to the control (H5V cells or LECs not treated with 4PYR) (Figure 6).

We can conclude that 4PYR could induce a cell-specific effect on the extravasation part of the metastatic cascade, regulating an endothelial barrier through its impact on eAdo accumulation and energy status. This would lead to the increased permeability of a barrier formed by the LEC cells. Such a change, when combined with the regulatory role of 4PYR in the metabolic interactions between cancer and endothelial cells, could explain the 4PYR-induced increase in lung metastases.

## 3. Discussion

4PYR was demonstrated to be highly accumulated in patients with metastatic BC [4], and it was indicated as being a potential metabolic marker of cancer progression and metastasis [45]. We demonstrated its role as an oncometabolite during the later stages of BC progression, increasing lung metastasis formation in the 4T1 experimental metastasis model. We have attributed this to the 4PYR-induced changes in extracellular adenine nucleotide metabolism and intracellular energy status in ECs, shifting catabolite patterns toward the accumulation of anti-inflammatory eIno, and leading to the increased permeability of lung ECs.

On the other hand, 4PYR did not affect primary tumor growth in the 4T1 orthotopic model, and it decreased the invasive potential of BC cells and their attachment to EC. However, the 4PYR-induced regulation of invasive potential was interdependent with the CD73-adenosine axis, and it was reversed by CD73 inhibition, while 4PYR reversed the impact of the A_3_AR agonist on ECM invasion by 4T1 cells. CD73-generated eAdo promotes tumor growth and progression, stimulating tumor neovascularization [31,46], immunosuppression [24], tumor cell proliferation, and invasive potential [47,48]. In a 4T1 cell culture, 4PYR stimulated eAdo accumulation, upregulating AMP hydrolysis but without a concomitant increase in the low activity of 4T1 cells in eAdo deamination. However, the 4T1 model is characterized by rapid primary tumor growth [49]. The fast development of hypoxia in rapidly growing tumors [50] and concurrent inflammation induce significant CD73 overexpression [51,52]. Together with the hypoxia-induced release of ATP from tumor cells, this stimulates massive eAdo accumulation [24] with CD73 as a rate-limiting enzyme in this process [13,53]. Thus, the modulatory effects of 4PYR in a rapidly growing solid tumor could be negligible, due to the overwhelming degree of signaling that is activated by accumulating eAdo.

We also observed that the inhibitory effect of 4PYR on the invasive potential of tumor cells was dependent on CD73 activity. CD73 inhibition itself did not affect ECM invasion by tumor cells, but it seemed to be a net effect of the opposite changes induced by the A_3_ and A_2A_ adenosine receptors. The effects induced by eAdo signaling are dependent on the type and number of adenosine receptors activated [14,47], with receptors A_1_, A_2A_, and A_3_ being activated at physiological adenosine levels, and the low-affinity receptor A_2B_ at higher pathological concentrations [54]. In the 4T1 model, tumor growth and metastasis formation were decreased by the inhibition of CD73 [55] or of adenosine deaminase [33], indicating that it is a deregulation of eAdo levels that can inhibit cancer progression. However, it still needs to be investigated as to whether 4PYR acts as an extracellular signaling molecule, i.e., an antagonist of A_3_AR, or whether it affects downstream signaling pathways. However, the changes in the invasive potential of tumor cells could be a net effect of the presence of both 4PYR and eAdo in the tumor microenvironment. We suggest that 4PYR, through its interdependent regulatory loop with the CD73-adenosine axis, might assist in fine-tuning its activity during cancer growth. It is also possible that the eAdo-eIno gradient formed between the hypoxic areas and the areas with the developing blood vessels and paracrine interactions between tumor cells and ECs.

4PYR increased the number of lung metastases by almost two-fold in the experimental metastasis model representing the cancer extravasation stage [56] when we simulated 4PYR accumulation in blood plasma [5] at this stage of cancer progression. 

While 4PYR inhibited the adhesion of tumor cells to ECs, this should not affect the formation of the lung metastases, as tumor cell arrest in the lungs is mainly due to the mechanical entrapment of tumor cells controlled by blood circulation patterns [57]. Furthermore, the detachment of cancer cells was shown to stimulate their clustering, a process limiting the formation of reactive oxygen species and enhancing tumor cell survival during metastatic spread [58]. It is also possible that 4PYR can stimulate the survival of tumor cells directly through the inhibition of the AMPD (AMP deaminase) pathway [19,59], resulting in SIRT1 stimulation [60,61].

Furthermore, inflammation is a crucial factor in the metastatic process, with circulating tumor cells being vulnerable to anti-metastatic immune surveillance, mainly by CD8+ T-cells and natural killer (NK) cells [62,63]. We observed that 4PYR can stimulate the switch from eAdo to eIno accumulation during tumor cell–EC interactions. Tumor cells that are deformed during the dissemination stage release pro-inflammatory ATP [39,64] and stimulate an increase in ATP release by ECs [36]. The rapid hydrolysis of ATP to anti-inflammatory eAdo suppresses T-cell and NK cell-mediated anti-tumor immunity [10,65]. Inosine can also potently suppress the immune response through the inhibition of the production of various pro-inflammatory cytokines, including TNF-α and IFN-γ [37,66,67]. However, eAdo is crucial in reducing inflammation-induced damage to the endothelial barrier [39], with a CD73 knockout leading to massive vascular leakage in response to hypoxia [66]. Such damage allows for the subsequent transmigration of tumor cells through the endothelial barrier [67,68]. The 4PYR-induced switch from eAdo to eIno accumulation can thus suppress the immune response without affecting damage to the ECs.

We have also demonstrated that 4PYR directly affects endothelial permeability, significantly increasing the permeability of the lung endothelial layer in vitro. 4PYR-induced endothelial toxicity is a result of the high energy requirements for its metabolization [17,18,19,69]. However, it induced disparate effects between ECs of different origins in correlation with the differences in their extracellular adenine nucleotide metabolism, energy status, and oxidoreductive potential. Such metabolic differences, together with the specific receptor patterns present on ECs [32], point towards the organ-specific role of 4PYR in the regulation of the metastatic process. The organ-specificity of 4PYR was postulated in our earlier research [17,19]. Such differences were also observed in different arteries from various species for NAD^+^-induced changes in the regulation of vascular tone through purine receptors [32].

Therefore, the 4PYR role seems to be important as an oncometabolite at the dissemination and colonization stage of cancer progression. It appears that it may also have a role in the organ tropism of circulating tumor cells, but this needs further study.

## 4. Materials and Methods

### 4.1. Reagents

4PYR (4-pyridone-3-carboxamide-1-β-D-ribonucleoside) and 2PYR (2-pyridone-5-carboxamide-1-β-D-ribonucleoside) were obtained by chemical synthesis at the Department of Biochemistry, Medical University of Gdansk [1]. AOPCP (adenosine 5′-*alpha,beta*-methylene diphosphate)(M3763), A_3_AR agonist IB-MECA (N^6^-(3-Iodobenzyl)adenosine-5′-N-methyluronamide, 1-Deoxy-1-[6-[((3-Iodophenyl)methyl)amino]-9H-purin-9-yl]-N-methyl-β-D-ribofuranuronamide)(I146), A_2A_AR agonist CGS-21680 (2-*p*-(2-Carboxyethyl)phenethylamino-5′-N-ethylcarboxamidoadenosine hydrochloride hydrate) (C141), A_1_AR agonist CCPA (2-Chloro-N^6^-cyclopentyladenosine)(C7938), Adenosine (A4036), AMP (01930), ATP (A26209), Calcein-AM (56496), Evans blue (E2129), HBSS (H9269), Mayer’s Hematoxylin solution (MHS32), Neutral Red (0.33% solution, N2889), Nicotinamide (NA) (72340), and phosphate-buffered saline (PBS)(P5493) were purchased from Sigma-Aldrich/Merck (Saint Louis, MO, USA). Nicotinamide Riboside (NR) was kindly provided by ChromaDex (Irvine, CA, USA). Accutase (25-058-Cl), sodium pyruvate (S8636), and penicillin/streptomycin (30-002-CI) were obtained from Corning (Corning, NY, USA). Fetal bovine serum (FBS) (10270106) was obtained from ThermoFisher (Waltham, MA, USA).

### 4.2. Cell Culture Conditions

H5V cell line (murine immortalized endothelial cells from the heart microvessels of C57BL/6 mice) [29] (kindly provided by Prof. A. Vecchi, Instituto Mario Negri, Milan, Italy) was cultured in DMEM medium, with high glucose (Corning, 10-017-CVR), supplemented with 2 mM L-glutamine, 1 mM sodium pyruvate, and 10% FBS. Murine 4T1 breast cancer cells (ATCC CRL-2539) were cultured in RPMI1640 medium with glutamine (Corning, 10-040-CVR), supplemented with 1 mM sodium pyruvate and 10% FBS. Human breast cancer cell lines (MDA-MB-231, ATCC HTB-26; T47D, ATCC HTB-133; MCF-7, ATCC HTB-22) were cultured in DMEM medium without sodium pyruvate (Corning, 10-017-CVR) and supplemented with 10% FBS. The immortalized cell line of murine embryonic fibroblasts NIH 3T3 (ATCC CRL-1658) was cultured in DMEM medium, with low glucose (Corning, 10-014-CVR) supplemented with 10% FBS. Cells were maintained in a 5% CO_2_ humidified atmosphere at 37 °C.

For the assays, cell cultures were supplemented with 100 μM 4PYR, 2PYR, NA, NR, 200 μM AOPCP (10 mm stock solutions in water), or 10 μM adenosine receptor agonists (5 mg/mL stock solutions in DMSO; 0.05% final DMSO concentration in cell culture). Solvents applied at appropriate concentrations were used as a vehicle for all controls.

### 4.3. Cell Viability Assay

Tumor or endothelial cells were seeded in a 96-well plate (3 × 10^4^ cells per well) and cultured overnight until they were 90–100% confluent. Cells were then serum-starved overnight, the medium was exchanged for fresh serum-free medium using serial dilutions of 4PYR, and the plate was incubated at 37 °C in a CO_2_ incubator for 24 h. After treatment, the medium containing the compound was removed, and 100 μL of Neutral Red solution (diluted 1:35 in serum-free medium) was added for 20 min. After incubation, cells were washed twice with PBS, and 100 μL of Sorensen’s buffer was added to each well, then the cells were lysed, and the dye was released through pipetting. Neutral Red absorbance was measured at 540 nm. As a reference blank, a well containing Sorensen’s buffer was used. The absorbance in wells containing cells not treated with 4PYR was assumed to indicate 100% viability.

### 4.4. Wound Healing Assay

4T1 cells were seeded in a 96-well plate (3 × 10^4^ cells per well) and cultured overnight until they were 90–100% confluent. Cells were then serum-starved overnight, and a linear wound was applied to the monolayer using a 200 μL micropipette tip. After washing the loose cells with PBS, cells were further cultured in serum-free medium, and supplemented, when indicated, with 100 μM 4PYR. Wounded areas were then imaged at 0 and 24 h using a Axiovert 200 microscope with 10 × A-Plan objective, AxioCam MRm, and AxioVision software (Carl Zeiss MicroImaging, Jena, Germany). The wound width was calculated using arbitrary units with the use of ImageJ software (NIH). The cell migration distance was determined by subtracting the width of the wound 24 h after its initial width at time 0. The values were plotted as the percentage of the wound closure, with the initial width set to 0%.

### 4.5. Migration and Invasion Assays

For the invasion assays, BioCoat Matrigel invasion chambers with 8.0 um PET membrane for a 24-well plate (Corning, 354480) were used in 24-well companion plates (Corning, 353504) forming a modified Boyden chamber. For the migration assays, transwell PET membrane inserts with a pore size of 8 µm (Corning, 353097) were used. Tumor cells were serum-starved overnight and added (2.5 mm 10^4^ for invasion, 1.5 × 10^4^ for migration) to the upper compartment in a 0.5 mL volume of serum-free medium, supplemented with 100 μM 4PYR, 2PYR, NA, NR, 200 μM AOPCP, or 10 μM adenosine receptor agonists, when indicated. To the lower compartment, 0.65 mL of conditioned medium from NIH/3T3 cell culture containing 10% of FBS was added as a chemoattractant for the invasion assay, or fresh medium supplemented with 10% FBS for the migration assay. After 24 h, cells were removed from the upper surface of the filter with cotton swabs. Invaded/migrated cells on the lower membrane surface were fixed in 4% paraformaldehyde, stained with Mayer’s Hematoxylin solution, and mounted on glass slides. Cells were then quantified by counting the number of stained cells from seven fields at predetermined areas on the membrane using an Olympus IX83 microscope with a 10 × UPLFLN10X2PH Universal Plan Fluorite objective, a DP74 color camera, and cellSens Imaging Software (Olympus, Tokyo, Japan). The mean values were calculated, and the data were presented as the percentage of control migration/invasion (vehicle-treated cells).

### 4.6. Cell Adhesion Assay

H5V cells were seeded in a 96-well plate (2.5 × 10^4^ cells per well) and cultured overnight to form a fully confluent monolayer, then serum-starved for 3 h. A subconfluent 4T1 tumor cell culture was serum-starved for 3 h in DMEM, then the tumor cells were suspended with Accutase and labeled with 2.5 µM Calcein-AM for 30 min in a CO_2_ incubator. After incubation, Calcein AM was washed out, and 4T1 cells were applied in serum-free DMEM (3 × 10^5^ cells/mL, 100 µL per well) on top of the endothelial monolayer. 4T1 or H5V cells were pretreated with 100 μM 4PYR during the serum starvation phase, where indicated. When only 4T1 cells were preincubated with 4PYR, it was carefully washed out, together with Calcein AM. When both 4T1 and H5V cells were pretreated with 4PYR, after washing out Calcein AM, 4PYR was supplemented to a final concentration of 100 μM during the assay. Tumor cells were allowed to interact with the endothelial cells for 15 min at 37 °C in a CO_2_ incubator (static adhesion phase), and then the unbound tumor cells were gently washed away. Whole wells were imaged using an Axiovert 200 epifluorescence microscope with a 20 × A-Plan objective, AxioCam MRm, and AxioVision software (Carl Zeiss MicroImaging, Jena, Germany). The number of adherent fluorescent cells present in each well was counted using ImageJ software (NIH). The mean values were calculated, and 100% adherence was assumed for the control wells (vehicle-treated cells).

### 4.7. Murine Model of 4T1 Breast Cancer

For the experimental lung metastasis model, 8–11-week-old female BALB/c mice purchased from the Tri-City Academic Experimental Animal Centre (Gdansk, Poland) were injected with 1.5 × 10^4^ of 4T1 breast cancer cells intravenously into a tail vein. For the orthotopic model, 8–11-week-old female BALB/c mice purchased from the Mossakowski Medical Research Centre (Warsaw, Poland) were injected with 3 × 10^5^ of 4T1 breast cancer cells into a mammary fat pad. Animals were randomly assigned to the groups: 4T1 (n = 10) and 4T1 + 4PYR (n = 10), and maintained for 28 days. 4PYR (100 mg/kg/24 h) or 0.9% NaCl (control) was administered subcutaneously every 12 h after the injection. After euthanasia (ketamine/xylazine, 100/10 mg/kg, i.p.), the lungs were excised, placed in Bouin fixative for at least 24 h, then the number of surface metastatic loci was counted with a stereomicroscope. The count was confirmed via histological analysis. The volume of the primary tumor was measured with calipers every 2–3 days, and changes in tumor volume were calculated. After euthanasia, the tumors were excised and weighted. Mice were housed in an individually ventilated cage (23 ± 1 °C, 40 ± 10% humidity) with a light/dark cycle of 12 h/12 h in environment-controlled rooms. Animals had unlimited access to water and a standard chow diet.

### 4.8. Isolation and Culture of Murine Lung Endothelial Cells

Four- to six-week-old C57BL/6 mice were anesthetized (ketamine/xylazine, 140/14 mg/kg), the thoracic cavity was exposed, and the lungs were removed. Isolated lungs were minced on a Petri dish and then incubated with 5 mL of collagenase A (2.5 mg/mL solution in 0.1% BSA in HEPES) in a 15 mL tube for 1 h in a 37 °C water bath with gentle shaking. The resulting tissue/cell suspension was filtered through a 70 μm strainer and washed twice with PBS. Cells were resuspended in an endothelial cell medium consisting of 4.5 g/L custom DMEM (PAN Biotech, Aidenbach, Germany, P04-03500S3), without L-glutamine and sodium pyruvate, with 3.7 g/L NaHCO_3_ and D-valine, and without L-valine, supplemented with ECGS (Sigma-Aldrich, Saint Louis, MO, USA, E2759), 10% FBS, 2 mM L-glutamine, and 1% penicillin/streptomycin (*v*/*v*) in T-25 tissue culture flasks, and incubated in a CO_2_ incubator. After reaching the specified density, the cells were sorted using mouse CD31 MicroBeads on a MACS column (Miltenyi Biotec, Bergisch Gladbach, Germany, 130-097-418), according to the manufacturer’s protocol. After sorting, cells were resuspended in an endothelial cell medium and passaged with the use of trypsin–EDTA. Cells below the fifth passage were used for experiments.

### 4.9. In Vitro Endothelial Permeability Assay

H5V cell line or isolated murine lung endothelial cells (LECs) were seeded on the upper surface of transwell PET membrane inserts (1 µm pore size, Corning, 353097) in a 24-well companion plate (Corning, 353504), with 2.5 × 10^5^ cells in 0.5 mL of DMEM medium or 1 mL of endothelial cell medium, respectively. The H5V cells were cultured overnight and the LECs were cultured for three days, to obtain a thin confluent monolayer. Then, the medium in the upper compartment was exchanged for a fresh medium supplemented with 100 μM 4PYR, and cells were incubated for 22 h. After incubation, the inserts were transferred into a receiver plate containing serum-free DMEM (equilibrated overnight in a CO_2_ incubator); 0.2 mL of Evans blue dye–BSA-bound solution (0.5% Evans blue dye in PBS with 0.1% BSA) was added into the upper compartment, and the plate was incubated for 30 min in a CO_2_ incubator. The extent of permeability was determined by measuring the absorbance of the BSA-bound Evans blue dye in the medium from the lower compartment of the receiver plate at a 610 nm wavelength [44]. Medium without Evans blue solution was used as a blank. The mean values were calculated, and 100% permeability was assumed for the control wells (vehicle-treated cells). Monolayer integrity was assessed after the experiment via the removal of the Evans blue dye solution, staining of cells with Mayer’s Hematoxylin solution, and the observation of the monolayer under a microscope for any alterations. Any monolayer in which cell detachment was visualized was discarded from the data set.

### 4.10. Tumor/Endothelial Cells Co-Culture

For the co-culture, 4T1 tumor cells were seeded into Transwell PET membrane inserts with a pore size of 1 µm (Corning, 353104) (6 × 10^4^ cells per insert), and H5V endothelial cells were seeded into a 24-well companion plate (Corning, 353504) (6 × 10^4^ cells per well). After 24 h of incubation, inserts containing 4T1 cells were transferred to a plate with H5V cells to form the transwell chamber. 4PYR or PBS (vehicle) (both 100 μM) were added to the upper chamber, and the cells were incubated for 72 h. Then, the inserts with the 4T1 cells were removed, and the H5V cells in a lower compartment were washed three times with PBS and used for the analysis of extracellular nucleotide metabolism. As a control, a single cell type was seeded into a Transwell chamber and analyzed after incubation.

### 4.11. Determination of Intracellular Nucleotide and 4PYR Derivatives Levels and Extracellular Nucleotide Metabolism

For the co-culture assay, cells were seeded into Transwell chambers as described above, and treated with 4PYR or PBS for 72 h. For the analysis of endothelial cells, H5V cell lines and LECs were seeded into 24-well plates (6 × 10^4^ cells per well) and cultured for 24 h, then the medium was replaced by fresh medium, and the cells were treated for 72 h with 100 μM 4PYR or PBS (vehicle). For the determination of the TNF effect on the H5V cell line, H5V cells were seeded in a 24-well plate (6 × 10^4^ cells per well), cultured for 72 h, then the medium was replaced by fresh medium, and the cells were treated for 6 or 24 h with 10 ng/mL of TNF-α or PBS. After treatment, for the determination of the enzymatic activity, cells were washed and incubated in HBSS with substrates for ectoenzymes. Adenosine, ATP, and AMP (all 50 μM) were sequentially added, with the medium being exchanged after each substrate. An inhibitor of adenosine deaminase-erythro-9-(2-hydroxy-3-nonyl) adenine (EHNA) was present at 5 μM concentration during the incubation with ATP and AMP, to block the conversion of adenosine to inosine. The medium was collected after 0 and 30 min of incubation at 37 °C in a CO_2_ incubator. Intracellular ATP, AMP, and NAD^+^ were also measured. To evaluate the influence of 4PYR on the concentration of intracellular nucleotides and 4PYR metabolism, cells were incubated with 100 μM 4PYR for 72 h. Afterward, cells were washed with HBSS. To extract the intracellular ATP, AMP, NAD^+^, 4PYMP, and 4PYRAD, 0.3 mL of 0.4 M HClO4 was added to each well, and the plates were frozen at −80 °C and thawed on ice twice. Next, cell extracts were collected and brought to pH 5.5–6 using 3M K_3_PO_4_. After centrifugation, the supernatants were analyzed via reversed-phase HPLC on a 15 cm/4.6 mm Hypersil BDS C18 3 lm column obtained from Thermo Fisher Scientific (28103-023006). An external calibration with pure compounds obtained through chemical synthesis was used for the quantitative analysis. Protein precipitates were dissolved in 0.5 mL 0.5 M NaOH and analyzed using the Bradford method for protein concentration (nmol/min/mg of protein) [19,70,71,72].

### 4.12. Statistical Analysis

Mean values were obtained from at least three separate experiments and reported as the mean (±SEM). Changes in the mean values of enzymatic activities or metabolite concentrations were evaluated using two-way analysis of variance (ANOVA) followed by Holm–Šídák post hoc, one-way ANOVA, and then followed by the Holm–Šídák post hoc test. Normality was assessed using the Shapiro–Wilk or Kolmogorov–Smirnov normality tests. For other experiments, the Mann–Whitney test for two unpaired groups of a non-Gaussian population was used. The exact value of n was provided for each type of experiment. A *p*-value < 0.05 was considered as being statistically significant.

## 5. Conclusions

We can conclude that 4PYR demonstrates significant potential as an oncometabolite for increasing lung metastasis formation. While 4PYR inhibits the invasive potential of tumor cells in vitro, in vivo, it is an accumulating eAdo that is generated by the flexible and adaptable CD73-adenosine axis, which has a crucial role in the regulation of tumor growth and invasion. 4PYR only seems to have a role in the fine-tuning of this axis without affecting the tumor growth. However, 4PYR can induce a switch from eAdo to eIno accumulation. It can suppress the immune response and inhibit the eAdo-regulated stabilization of the endothelial barrier. Together, with the direct endothelial toxicity of 4PYR through the downregulation of cellular energetics, this results in the increased permeability of the lung endothelial cells. Therefore, the 4PYR role seems to be important as an oncometabolite at the dissemination and colonization stage of cancer progression. We can also suggest upon its role in the organ tropism of circulating tumor cells, due to its disparate effects between ECs of different origins.

## Data Availability

Not applicable.

## Supplementary Materials

The supporting information can be downloaded at: https://www.mdpi.com/article/10.3390/ijms23105774/s1.
