# Supplementary material for: 4-Pyridone-3-carboxamide-1-β-D-ribonucleoside (4PYR)—A Novel Oncometabolite Modulating Cancer-Endothelial Interactions in Breast Cancer Metastasis
Source: Int J Mol Sci. 2022 May 21;23(10):5774. doi: 10.3390/ijms23105774 (PMC9145394; doi:10.3390/ijms23105774)
Supplement: Supplementary file 1 [file ijms-23-05774-s001.zip › ijms-1691900-supplementary.pdf]

Supplementary Data

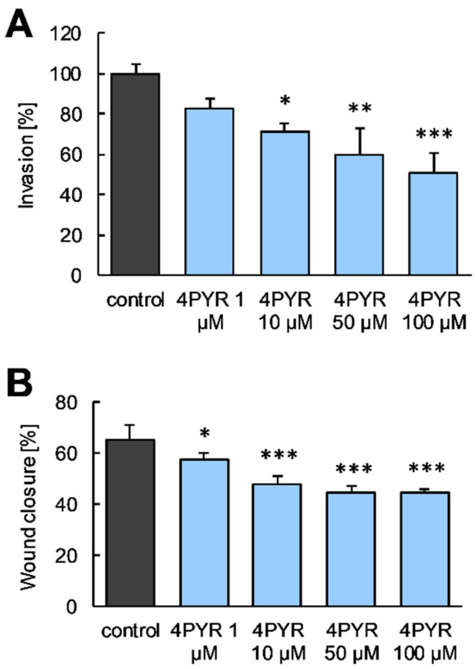

Figure S1. 4PYR-induced the decrease in invasion and migration of 4T1 breast cancer cell line *in vitro* is dose-dependent. (A) 4PYR inhibited Matrigel invasion of 4T1 cells at a concentration range from 10 to 100 µM, while it had no effect at 1 µM. After overnight serum starvation, cells were seeded in a transwell invasion chamber and treated with serial dilutions of 4PYR, as indicated on a graph, for 24 hours. The percentage of cells that invaded across the filter was counted by Mayer's Hematoxylin staining after fixation. The graph represents the mean ± SEM of four independent experiments. *p<0.05, **p<0.01, ***p<0.001 vs. control (non-treated cells). **(B)** 4PYR inhibits collective migration of 4T1 cells at a concentration range from 1 to 100 µM. Cells were seeded in a 96-well plate, and after overnight serum-starvation, a linear wound was applied. The percentage of wound closure was calculated by subtracting the width of the wound after 24 hours from its initial width at time 0 (demonstrated as bars on representative images). The graph represents the mean ± SEM of four independent experiments. *p<0.05, ***p<0.001 vs. control (non-treated cells).

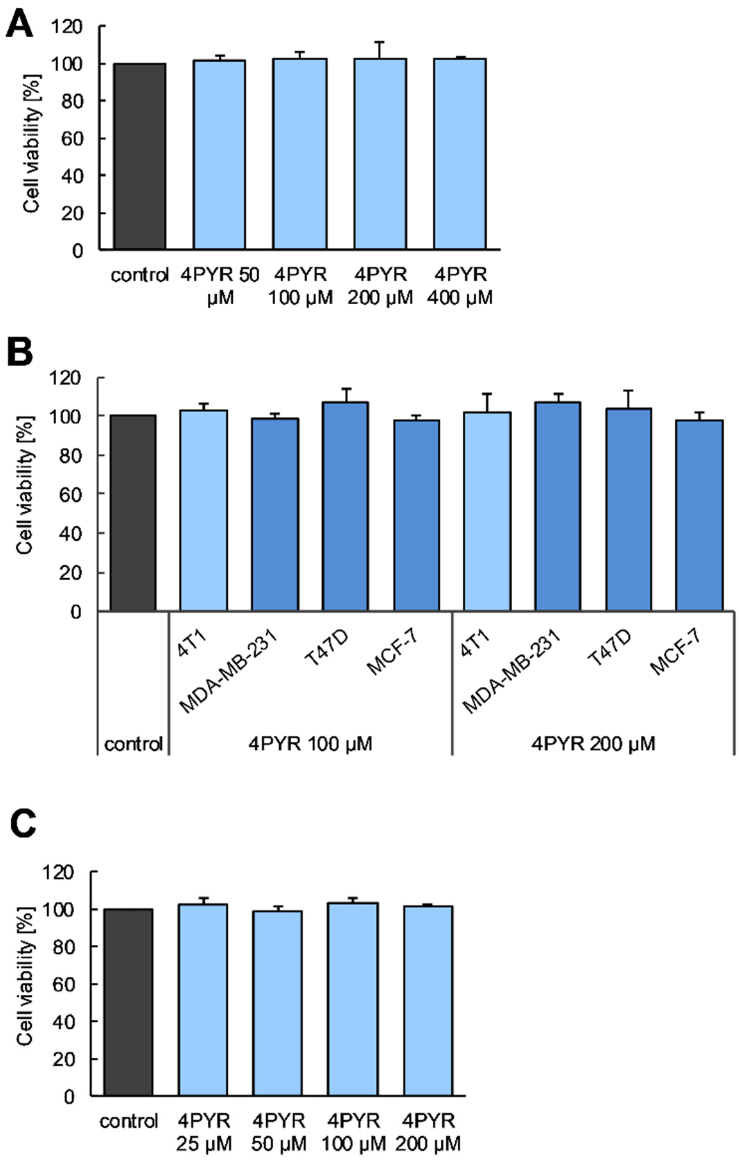

Figure S2. 4PYR does not affect the viability of analyzed cells, both murine and human tumor cell lines, and an endothelial cell line. (A) 4PYR did not affect the viability of 4T1 murine breast cancer cell line at the concentration range of 50-400 µM, **(B)** 4PYR did not affect the viability of human breast cancer cell lines (MDA-MB-231, T47D, and MCF-7) at a concentration of 100 and 200 µM, comparable to 4T1 cell line, **(C)** 4PYR did not affect the viability of H5V murine endothelial cell line at the concentration range of 25-200 µM. Viability was analyzed with a Neutral Red uptake assay *in vitro*. Mean ± SEM, n=3.
